# Genome-wide association study with 1000 genomes imputation identifies signals for nine sex hormone-related phenotypes

**DOI:** 10.1038/ejhg.2015.102

**Published:** 2015-05-27

**Authors:** Katherine S Ruth, Purdey J Campbell, Shelby Chew, Ee Mun Lim, Narelle Hadlow, Bronwyn GA Stuckey, Suzanne J Brown, Bjarke Feenstra, John Joseph, Gabriela L Surdulescu, Hou Feng Zheng, J Brent Richards, Anna Murray, Tim D Spector, Scott G Wilson, John RB Perry

**Affiliations:** 1 Genetics of Complex Traits, University of Exeter Medical School, University of Exeter, Exeter, UK; 2 Endocrinology and Diabetes, Sir Charles Gairdner Hospital, Nedlands, Australia; 3 Pathwest Laboratory Medicine WA, Nedlands, Australia; 4 School of Medicine and Pharmacology, University of Western Australia, Nedlands, Australia; 5 Department of Epidemiology Research, Statens Serum Institut, Copenhagen, Denmark; 6 Department of Twin Research and Genetic Epidemiology, King's College London, London, UK; 7 Department of Medicine, Human Genetics, McGill University, Montreal, Canada; 8 Lady Davis Institute, McGill University, Montreal, Canada; 9 MRC Epidemiology Unit, Institute of Metabolic Science, Addenbrooke's Hospital, University of Cambridge, Hills Road, Cambridge, UK

## Abstract

Genetic factors contribute strongly to sex hormone levels, yet knowledge of the regulatory mechanisms remains incomplete. Genome-wide association studies (GWAS) have identified only a small number of loci associated with sex hormone levels, with several reproductive hormones yet to be assessed. The aim of the study was to identify novel genetic variants contributing to the regulation of sex hormones. We performed GWAS using genotypes imputed from the 1000 Genomes reference panel. The study used genotype and phenotype data from a UK twin register. We included 2913 individuals (up to 294 males) from the Twins UK study, excluding individuals receiving hormone treatment. Phenotypes were standardised for age, sex, BMI, stage of menstrual cycle and menopausal status. We tested 7 879 351 autosomal SNPs for association with levels of dehydroepiandrosterone sulphate (DHEAS), oestradiol, free androgen index (FAI), follicle-stimulating hormone (FSH), luteinizing hormone (LH), prolactin, progesterone, sex hormone-binding globulin and testosterone. Eight independent genetic variants reached genome-wide significance (*P*<5 × 10^−8^), with minor allele frequencies of 1.3–23.9%. Novel signals included variants for progesterone (*P*=7.68 × 10^−12^), oestradiol (*P*=1.63 × 10^−8^) and FAI (*P*=1.50 × 10^−8^). A genetic variant near the *FSHB* gene was identified which influenced both FSH (*P*=1.74 × 10^−8^) and LH (*P*=3.94 × 10^−9^) levels. A separate locus on chromosome 7 was associated with both DHEAS (*P*=1.82 × 10^−14^) and progesterone (*P*=6.09 × 10^−14^). This study highlights loci that are relevant to reproductive function and suggests overlap in the genetic basis of hormone regulation.

## Introduction

Studies have suggested that genetic factors contribute significantly to population variance in sex hormone levels, however, few associated genetic variants and genes have been identified to date.^1^ As well as playing an important role in reproduction, variations in sex hormone levels can have wider implications for health and disease. Reproductive functions include control of the menstrual cycle, spermatogenesis, steroidogenesis and lactation, and sex hormone levels have been implicated in breast cancer, cardiovascular disease, osteoporosis, type 2 diabetes and ageing.^2, 3, 4, 5^ Circulating levels of sex hormones are limited by sex hormone-binding globulin (SHBG), which is a glycoprotein that binds and transports oestradiol, testosterone and dehydroepiandrosterone (DHEA) to a lesser extent.^6^

GWAS have been performed for dehydroepiandrosterone sulphate (DHEAS), SHBG, follicle-stimulating hormone (FSH), luteinizing hormone (LH), oestradiol and testosterone.^2, 4, 7, 8, 9, 10^ The largest of these GWAS was in over 28 000 males and females and identified 12 loci associated with differences in SHBG levels, including four loci with sex-specific genetic effects and considerable allelic heterogeneity at the *SHBG* gene locus.^7^ A recent study in 3495 Chinese men has identified a novel locus associated with oestradiol and FSH levels, and a further novel locus for oestradiol.^10^ A GWAS of total testosterone in males identified three loci, including two in the *SHBG* gene, that were also associated with SHBG levels.^9^ In an analysis of males and females combined, eight loci associated with DHEAS were identified, of which several were associated with changes in gene expression levels in pathways linked to ageing.^4^ GWAS studies of sex hormone-related phenotypes have explained less than 10% of variance in oestradiol and SHBG, and less than 5% of variance in testosterone, DHEAS and FSH.^4, 7, 9, 10^

In this study, we performed a 1000 Genomes imputed GWAS to identify novel genetic variants in sex hormone-related phenotypes where either GWAS has not yet been performed or has not been performed at 1000-Genomes-density variant coverage.

## Materials and Methods

### Study population

The study included up to 2913 individuals of European ancestry from the Twins UK study with genotype and phenotype data.^11^ Twins UK is a supported access resource with all data access requests overseen by the Twins UK Resource Executive Committee. All studies have ethical approval from the Guy's and St Thomas' Ethics Committee (for further information, see http://www.twinsuk.ac.uk/data-access/). The Twins UK cohort is 51% monozygotic and 49% dizygotic.^11^ Individuals included in the analysis were mostly females, however, a small number of males (maximum of 294) were also included (Supplementary Table 1). Individuals who were pregnant or currently receiving hormone replacement therapy or oral contraceptive treatments were excluded from the analysis. Twins UK samples have been included in previous GWAS of DHEAS and SHBG.^4, 7^

### Phenotypes

Plasma levels of DHEAS, FSH, LH, oestradiol, progesterone, prolactin, SHBG and testosterone were measured by commercial ElectroChemiLuminescent immunoassays on a Modular Analytics E170 analyser (Roche Diagnostics GmbH, Mannheim, Germany) using the prescribed assay calibrators and performed according to the manufacturer's protocol. The specific assays used were: DHEA-S (03000087; CalSet 03000095), FSH (11775863; CalSet II 03032680), LH (11732234; CalSet II 03561097), Estradiol II (03000079; CalSet II 03064921), Progesterone II (12145383; CalSet 12145391), Prolactin II (03203093; CalSet 03277356), SHBG (03052001: CalSet 03052028) and Testosterone II (05200067; CalSet II 05202230). Details of the immunoassays are provided in the Supplementary Information. Free androgen index (FAI) was calculated as (testosterone/sex hormone-binding globulin) × 100.^12^ Individual sex hormone measures were fitted in a regression model against age, sex, BMI, phase of menstrual cycle (for females, as a categorical variable), menopausal status, after which the residuals were transformed to approximate a normal distribution (either through log, square-root or inverse rank normal transformation) and outliers more than four standard deviations from the mean were removed. Single nucleotide polymorphism (SNP) beta estimate effect sizes are quoted as a per-allele standard deviation change in the covariate-adjusted transformed residuals. The number of individuals included in the analysis of each hormone was 2899 for DHEAS, 2906 for oestradiol, 2699 for FAI, 2885 for FSH, 2881 for LH, 2865 for prolactin, 2689 for progesterone, 2913 for SHBG and 2657 for testosterone (differences in the numbers for FAI and testosterone are accounted for by removal of outliers prior to inclusion in the GWAS).

### Genotypes

Genotyping of the TwinsUK dataset was done with HumanHap300, HumanHap610Q, HumanHap1M Duo and HumanHap1.2M Duo 1M arrays. Imputation was done in two datasets (*n*=2040 from the HumanHap300 array; *n*=3614 from the HumanHap610Q, HumanHap1M Duo and 1.2M Duo 1M arrays) which were then merged with GTOOL. We performed imputation for Twins UK study subjects based on 1000 Genomes data as described previously.^13^ This involved estimating the phase of contiguous variants in the subjects using the haplotypes calculated from the 1000 Genomes Project consisting of 1094 individuals and 2188 haplotypes and the program MACH 1.0.16. The variants in the build-37 November 2010 release of 1000 Genomes (Phase 1-α interim) were imputed into the phased haplotypes using MINIMAC. This resulted in 37 426 733 imputed SNPs. We excluded SNPs that were imputed with an *r*^2^_imp_<0.5. This left 10 879 115 SNPs and after filtering for minor allele frequency (MAF)>0.01 the number fell to 7 879 351. We used a multi-ethnic reference panel that included 381 Europeans (including 98 Tuscans), 181 Americans, 246 Africans and 286 Asians to improve the quality of imputation, particularly at lower frequency variants.^14^

### Statistical analysis

We performed a linear mixed-model GWAS analysis for each of the hormones using the program GEMMA,^15^ which is capable of accounting for relatedness of the study subjects when applicable, as well as population stratification and cryptic relatedness. Association statistics using the score test were calculated for 7 879 351 autosomal SNPs passing a MAF filter of 0.01 and an imputation quality score of 0.5.

The Bonferroni-corrected *P*-value for the number of SNPs tested across nine traits was *P*<7 × 10^−10^; however, this is likely to be conservative given that many SNPs are unlikely to be independent and there are Bayesian arguments for less conservative *P*-values.^13^ Hence, we considered independent significant SNPs to be those with *P*<5 × 10^−8^ and more than 1 Mb away from another significant SNP. The UCSC Genome Browser and Locus Zoom were used to identify genes in the regions where significant SNPs were identified.^16, 17^ SNAP, HaploReg v2, Locus Zoom and Ensembl Biomart were used to identify HapMap proxies for the 1000 Genomes signals, with linkage disequilibrium evaluated in the 1000 Genomes Phase I CEU population.^17, 18, 19, 20^ We analysed expression quantitative trait loci data to identify associations between SNPs associated with variation in hormone levels and expression levels of nearby genes in the Multiple Tissue Human Expression Resource (MuTHER).^21^ Functional annotation of SNPs in strong linkage disequilibrium with the significant signals (*r*^2^>0.8) was performed using wANNOVAR, GWAVA and HaploReg v2.^20, 22, 23^

## Results

### Hormone phenotypes are correlated

There were strong correlations between three groups of phenotypes included in our study (Table 1): (i) FAI, SHBG and testosterone; (ii) progesterone, DHEAS and testosterone; and, (iii) FSH and LH. FAI was positively correlated with testosterone (*r*=0.69) and negatively correlated with SHBG (*r*=−0.61), as would be expected because FAI is a calculated index of the amount of androgen not bound by SHBG. Testosterone and SHBG were not correlated (*r*=0.04). Progesterone was positively correlated with DHEAS (*r*=0.60) and, to a lesser extent, testosterone (*r*=0.44). As a result of the correlation with testosterone, progesterone was also correlated with FAI (*r*=0.39). DHEAS was also positively correlated with testosterone (*r*=0.55) and, as a result, FAI (*r*=0.52). There was a strong positive correlation between FSH and LH (*r*=0.63). Though the other correlations were smaller, oestradiol was positively correlated with testosterone (*r*=0.22) and was negatively correlated with FSH (*r*=−0.24).

### Three novel association signals

**Figure 1:**
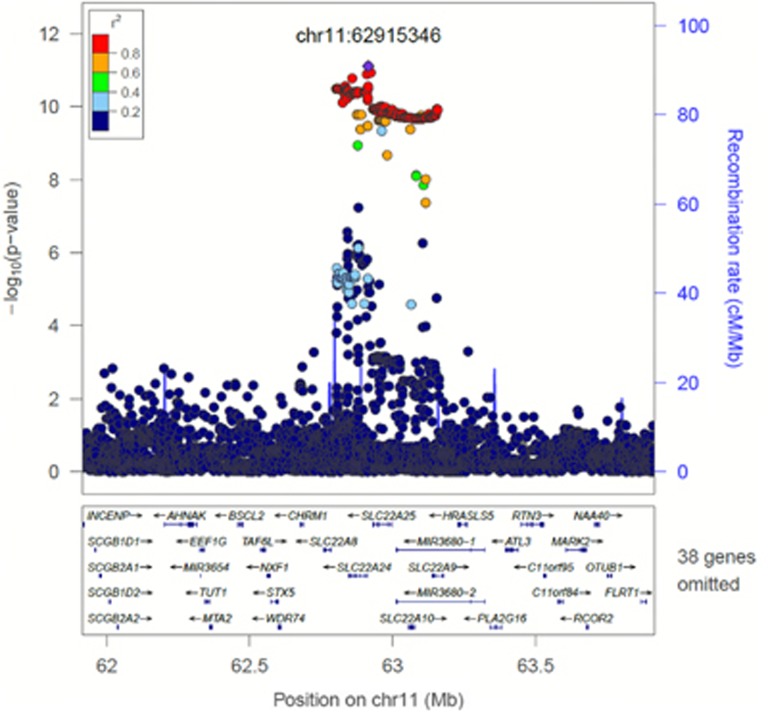
SNPs within 1 Mb of the significant signal for progesterone on chromosome 11 (rs112295236; chr11.hg19:g.62915346C>G). Note: Not all genes are shown. Linkage disequilibrium is based on 1000 Genomes Nov 2010 EUR.

We identified new signals for progesterone, oestradiol and FAI (Table 2). The signal for progesterone (rs112295236, *P=*7.68 × 10^−12^, MAF=0.06) was identified in an intergenic region of chromosome 11 (Figure 1). We searched for associations of the progesterone SNP with other traits that might be influenced by progesterone levels, but we found no evidence for association with other phenotypes (*P*>0.05 in published GWAS of age at menopause, early menopause, age at menarche, BMI, height, type 2 diabetes and glycaemic traits, endometriosis, and birth weight (maternal and foetal genotype)) or in a GWAS of pre-term delivery (five gestational age/pre-term delivery traits for mother's and child's genotype, unpublished data). The signals for FAI and oestradiol were located at 16q12.2 (rs117145500, near *LOC643714*, *P*=1.50 × 10^−8^, MAF=0.06) and 12p13.31 (rs117585797, in *ANO2*, *P*=1.63 × 10^−8^, MAF=0.01), respectively, and demonstrated no association with other tested complex traits.

### Five signals in known regions

We identified two signals that replicated previous associations, and a further three that have been reported previously, but for other phenotypes (Table 2). The signals for SHBG (rs1641549, near *SHBG* gene, *P*=1.21 × 10^−15^, MAF=0.24) and DHEAS (rs148982377, in *ZNF789*, *P*=1.82 × 10^−14^, MAF=0.04) have both been reported previously^4, 7, 9^ (Supplementary Table 2). The significant associations for FSH (rs11031005; *P*=1.74 × 10^−8^, MAF=0.13) and LH (rs11031002; *P*=3.94 × 10^−9^, MAF=0.12) are highly correlated SNPs (*r*^2^=0.79), residing in an intergenic region near the *FSHB* gene, and are in linkage disequilibrium with a published variant for menopause age (rs12294104) (*r*^2^=0.37 for FSH (rs11031005) and *r*^2^=0.45 for LH (rs11031002)).^24^ The strongest association for progesterone (rs34670419; *P=*6.09 × 10^−14^, MAF=0.04) was located on chromosome 7 in the 3' untranslated region of *ZKSCAN5* (Table 2; Supplementary Figure 1), a locus previously reported as associated with DHEAS levels.

### Two pairs of phenotypes have common signals

We identified overlaps between signals for FSH/LH and progesterone/DHEAS (association results for significant signals in all phenotypes are in Supplementary Table 3). The signals for FSH and LH were in linkage disequilibrium (*r*^2^=0.79), and the most significant SNP for FSH also reached genome-wide significance for LH; however, the direction of effects was the opposite of that expected by the phenotypic correlation (ie, levels of FSH and LH are positively correlated, though the minor allele decreased FSH and increased LH (Supplementary Table 3). The strongest signal for progesterone on chromosome 7 (rs34670419) was in linkage disequilibrium with the signal for DHEAS (*r*^2^=1), with allelic effects consistent with the expected phenotypic correlation (Supplementary Table 3).

### Overlap between DHEAS and progesterone variants

To investigate the genetic overlap of DHEAS and progesterone further, we tested whether five published variants for DHEAS (identified prior to conditional analysis) were associated with progesterone levels in our data.^4^ One of these variants reached genome-wide significance in our progesterone data (rs11761528, *P=*3.34 × 10^−8^) (Supplementary Table 4), and was at the same locus as our strongest progesterone signal (rs34670419, chr7:99130834). The published signal was not the strongest signal in our analysis though it was 12 kb from and in moderate linkage disequilibrium with our top chromosome 7 progesterone signal (*r*^2^=0.49). However, four of the five published variants were consistent in direction of effect (*P*=0.19) and two were nominally significant after Bonferroni correction (Supplementary Table 4). In addition, we investigated whether our two progesterone signals were significant in other published DHEAS meta-analysis data by looking up our two progesterone signals in data from Zhai *et al.*^4^ A proxy for our progesterone signal on chromosome 7 (*r*^2^=0.58) was strongly associated with DHEAS (*P*=2.34 × 10^−34^) and our progesterone signal on chromosome 11 showed weak evidence of association (*P=*1.53 × 10^−4^) (Supplementary Table 5).^4^

### FSH and LH signals overlap with a menopause locus

There was evidence of overlap between the signals for FSH and LH with a variant for menopause age on chromosome 11 (Supplementary Tables 6–9). There was moderate linkage disequilibrium between the signals for FSH and LH and a published variant for menopause age (rs12294104), with *r*^2^=0.37 for FSH (rs11031005) and *r*^2^=0.45 for LH (rs11031002), *P*= 3.02 × 10^−7^ for FSH and *P*=6.25 × 10^−7^ for LH.^24, 25^ None of the other published menopause or menarche variants were associated with FSH or LH at Bonferroni-corrected *P*<0.05.^24, 26^

### Identification of potentially causal candidate genes

**Figure 2:**
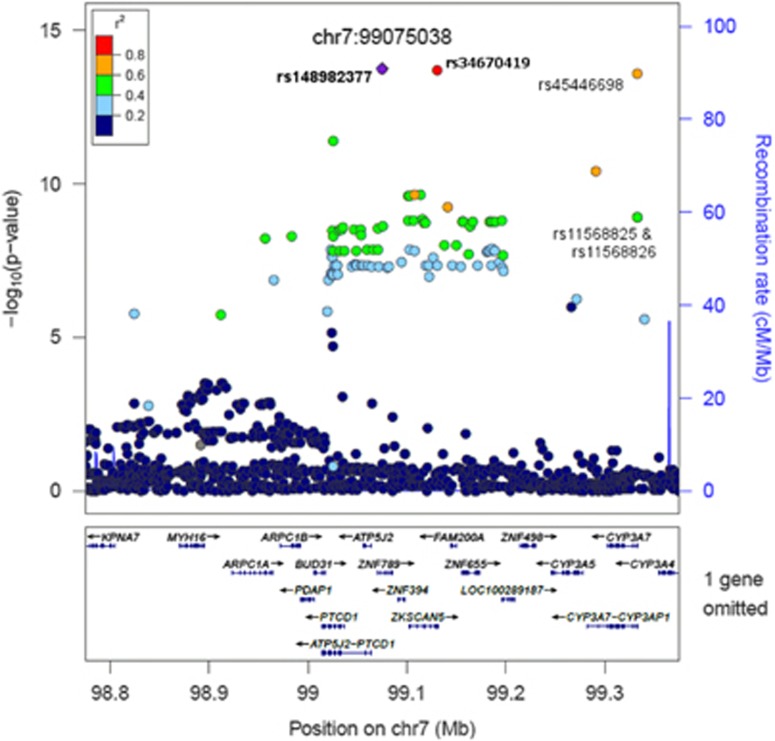
SNPs within 300 kb of the significant signal for DHEAS on chromosome 7 (rs148982377; chr7.hg19:g.99075038 T>C). SNPs indicated are the strongest progesterone signal (rs34670419 (chr7.hg19:g.99130834G>T)) and those in the *CYP3A7* promoter polymorphism that were identified in this analysis (rs45446698 (chr7.hg19:g.99332948 T>G), rs11568825 (chr7.hg19:g.99332986 A>C) and rs11568826 (chr7.hg19:g.99332978 A>T)). Note: Not all genes are shown. Linkage disequilibrium is based on 1000 Genomes Nov 2010 EUR.

The signal for SHBG (rs1641549) was 38 kb from the protein-coding gene *SHBG*. Four other signals were in linkage disequilibrium with known polymorphisms that have functional consequences: The signals for DHEAS (rs148982377) and progesterone (rs34670419) were in linkage disequilibrium with three SNPs (rs45446698, rs11568825 and rs11568826; *r*^2^>0.4 for all) that are part of a polymorphism in the promoter of *CYP3A7*, which is known to be associated with lower DHEAS levels (Figure 2). All three of these SNPs were genome-wide significant for DHEAS (*P*=2.49 × 10^−14^ for rs45446698; *P*=1.18 × 10^−9^ for rs11568825 and rs11568826) and one was genome-wide significant for progesterone (rs45446698, *P*=7.7 × 10^−11^), with the other two almost reaching significance (*P*<5 × 10^−7^). The top signals for LH and FSH were within 38 kb of, and in moderate linkage disequilibrium with, a known polymorphism (rs10835638) in the promoter of *FSHB* (rs11031005, *r*^2^=0.62; rs11031002, *r*^2^=0.74) (Supplementary Figure 2).^27^ Although the published *FSHB* promoter polymorphism was not the strongest signal in this region, it was genome-wide significant for association with LH in our data (*P=*4.84 × 10^−9^), and nearly significant for FSH (*P=*2.31 × 10^−7^) (Supplementary Table 11). Other candidate genes were identified by a search of a 300 kb region around each signal and are listed in Supplementary Table 10. Functional annotation of the signals with wANNOVAR did not reveal any additional likely causative variants.

## Discussion

In this study of nine sex hormone-related phenotypes, we identified three new signals and two pairs of phenotypes with a common signal. Four of the eight significant signals reached a conservative significance level of *P*<7 × 10^−10^, including the new signal for progesterone. This is the first published GWAS for the hormones progesterone, prolactin and the hormone measure FAI, and we identified genetic associations for all except prolactin. As we are not aware of any other genotyped cohorts with measurements for progesterone and FAI, we have been unable to replicate these findings. The hormones DHEAS, FSH, LH, SHBG, testosterone and oestradiol have been included in previously published GWAS, and we have compared our results with these existing data.^2, 4, 7, 8, 9, 10^ This study is, to our knowledge, one of the first published GWAS of hormones using 1000 Genomes Phase I imputed data. Three of the signals we identified were low frequency (less than 5%) and had large effect sizes (more than 50% change relative to standard deviation). In addition to identifying novel signals, we also observed two previously identified signals, demonstrating that true signals can be identified for these traits even in modest sample sizes.

The progesterone signal (rs112295236) that we identified on chromosome 11 was upstream of *SLC22A9*, which codes for an organic anion transporter OAT7 found in the liver. OAT7 is involved with the transport of DHEAS and oestrone-3-sulphate in exchange for butyrate, and is thought to be important for the release of oestrogen-3-sulphate into the blood.^28^ We did not find evidence to support this hypothesis in expression data, however, such data are only currently available for a limited range of tissues (skin, lymphoblastoid cell lines and adipose), not including ovary, which is the main site of progesterone synthesis. However, there was evidence of an association between this progesterone signal and DHEAS levels in data from a published GWAS, albeit at sub-genome-wide significance levels,^4^ and DHEAS and progesterone were both strongly correlated in our study (*r*=0.60). We postulated that the progesterone variant may be associated with phenotypic outcomes, such as offspring birth weight, or age at menopause, but we found no associations in data from other studies. Of course the effect size of our variant was relatively small and we may be underpowered to detect additional phenotypic associations.

The variant associated with decreased FAI in our analyses (rs117145500) showed some evidence of association with increased SHBG and decreased DHEAS, consistent with the role of DHEA in testosterone synthesis and the effect of SHBG on the amount of free androgens. This was supported by a negative correlation of FAI with SHBG in our data (*r*=−0.61) and positive correlation with DHEAS (*r*=0.55).

We identified a locus showing borderline significant association with oestradiol that requires further replication. The genetic variant associated with oestradiol (rs117585797) is a low frequency variant (MAF=0.013) with a large effect size, and is in an intron of the *ANO2* gene on chromosome 12. A previous GWAS of oestradiol levels in postmenopausal women did not identify any genetic variants reaching genome-wide significance in this region, though this may have been underpowered to detect this signal.^2^ Two other genes are present within the same chromosomal region (*vWF* and *CD9*), though there is not strong evidence to support one as a more likely candidate over the other. *VWF* (47 kb away) codes for the von Willebrand factor (vWF) protein which is involved in haemostasis, aiding platelet adhesion and preventing factor VIII degradation. Oestradiol has been shown to increase vWF production by endothelial cells *in vitro*,^29^ and in post-menopausal women, oral oestrogen treatment has been shown to increase vWF (though transdermal treatment did not show this effect).^30^ *CD9* (298 kb away) is a widely expressed cell surface molecule that has been shown to be required for sperm–egg fusion in mice.^31^

We provide evidence for overlap in the genetic regulation of two pairs of hormones whose levels are strongly correlated: FSH and LH (*r*=0.63); and progesterone and DHEAS (*r*=0.6). The FSH/LH variants were in linkage disequilibrium with a functional polymorphism (-211 G→T) in a progesterone response element of the promoter of the *FSHB* gene, which codes for the beta polypeptide of FSH. In females, FSH receptors are reported in endometrium^32^ and in granulosa cells. In granulosa cells, stimulation with FSH augments the expression of LH receptors.^33^ *In vitro* studies have demonstrated that the allele in linkage disequilibrium with the effect alleles in our study reduces levels of FSHB expression.^34, 35^ Previous studies provide conflicting data regarding the direction of effect of this polymorphism on FSH and LH levels.^27, 36, 37^ In our study, despite a positive overall correlation between FSH and LH levels, the genetic variants were negatively associated with FSH and positively associated with LH. Although this appears counter-intuitive, a similar situation is seen for other traits, for example, genetic variants that increase fasting glucose are not always risk factors for type 2 diabetes.^38^ Thus, the relationship between FSH and LH is complex and will involve additional genetic and non-genetic factors. A previous GWAS including FSH and LH did not find this signal, though this study was in Chinese men^10^ compared with our study of mainly female Europeans. Our study sheds light on part of the biological interaction between FSH and LH, but further variants need to be identified to understand this more fully.

The second pair of hormones with overlap in genetic regulation was DHEAS and progesterone. We identified a signal for progesterone on chromosome 7 in linkage disequilibrium with a known signal for DHEAS, plus evidence for association of progesterone levels with six of eight known DHEAS SNPs and of DHEAS levels with our newly identified chromosome 11 progesterone SNP. Both progesterone and DHEAS are steroid hormones that have a common precursor in their synthesis pathways (pregnenolone), but neither are directly synthesised from each other.^3^ These hormones were positively correlated in our data (*r*=0.60). The top signal for DHEAS in our study was rs148982377, which tagged a polymorphism in the promoter of *CYP3A7*, which was also in linkage disequilibrium with the top progesterone signal. *CYP3A7* has a progesterone response element that is thought to regulate expression during pregnancy.^39, 40, 41^ In the same region is the *CYP3A4* gene, which codes for a cytochrome P450 enzyme that metabolises progesterone, DHEAS, oestrone and testosterone.^42^

The main limitation of our study is the absence of suitable replication cohorts for the hormone measures progesterone, oestradiol and FAI. Half of the signals reached a conservative, Bonferonni-adjusted significance level of *P*<7 × 10^−10^, giving us confidence in the findings for progesterone, DHEAS and SHBG. Although the signals for FAI, FSH, LH and oestradiol reached a less conservative significance threshold, there are strong arguments for the validity of less stringent *P*-values in 1000 Genomes imputed GWAS.^13^ Further large studies are required to enable validation of our results. Such studies should also allow detection of new signals because the power of our study was limited by sample size and by the relatedness of the study individuals. Approximately 10% of our cohort consisted of males, and thus we will have been more likely to detect genetic effects in females than males, though our ability to detect genetic variants affecting both sexes should not have been affected. The cohort used in our study was of European ancestry and as such our findings will need to be replicated in other ethnic populations. Further GWAS studies of these hormones may benefit from an increase in power by implementing newly emerging multi-variate methods which would take account of correlations between the hormones.^43^ Any additional studies should also consider the need to directly quantify the effect of the variants, as it is difficult to relate the adjusted and transformed hormone measure that we used for our analysis to actual physiological changes. Further studies are required to address these issues.

In this GWAS of nine sex hormone-related phenotypes, we were able to detect three new signals (oestradiol, FAI and progesterone traits), two pairs of signals overlapping with other traits (FSH/LH and progesterone/DHEAS) and two signals seen before (DHEAS and SHBG traits). We have demonstrated potential overlap in the genetics of hormone regulation, as might be expected from common pathways in hormone synthesis. As well as the overlap in the top signals for DHEAS and progesterone, and FSH and LH, there were other variants associated with more than one hormone at lower significance levels, suggesting further commonality in hormone regulation. We identified novel genetic variants and potential overlap in the genetic basis of hormone regulation that will inform future studies, not only of hormones but also of common diseases, ageing and reproductive lifespan that include sex related hormones on the aetiological pathway.

## Figures and Tables

**Table 1:** Correlation coefficients between the sex hormone-related phenotypes

		*Oestradiol*	*Prolactin*	*DHEAS*	*SHBG*	*Progesterone*	*LH*	*FSH*	*Testosterone*
									
		*log*_*10*_ *(Oestradiol residuals)*	*log*_*10*_ *(Prolactin residuals)*	*square root (DHEAS residuals)*	*ln (SHBG residuals)*	*log*_*10*_ *(Progesterone residuals)*	*LH residuals*	*inverse normal (FSH residuals)*	*Testosterone residuals*
Oestradiol	log_10_ (Oestradiol residuals)	1	—	—	—	—	—	—	—
Prolactin	log_10_ (Prolactin residuals)	**0.09**	1	—	—	—	—	—	—
DHEAS	square root (DHEAS residuals)	**0.09**	−0.01	1	—	—	—	—	—
SHBG	ln (SHBG residuals)	**0.08**	**0.06**	**−0.15**	1	—	—	—	—
Progesterone	log_10_ (Progesterone residuals)	**0.17**	**0.04**	**0.60**	**−0.06**	1	—	—	—
LH	LH residuals	0.00	**0.14**	0.03	0.03	0.04	1	—	—
FSH	inverse rank normal transformed (FSH residuals)	**−0.24**	**0.08**	0.01	−0.01	0.00	**0.63**	1	—
Testosterone	Testosterone residuals	**0.22**	0.03	**0.55**	**0.04**	**0.44**	**0.06**	−0.01	1
FAI	FAI residuals	**0.11**	0.00	**0.52**	**−0.61**	**0.39**	0.02	−0.01	**0.69**

Note: Values in bold are significant at *P*<0.05.

**Table 2:** Variants significantly associated with hormone levels (*P*<5 × 10^−8^)

*Hormone*^a^	*Chr-position*^b^	*SNP id*	*Imputation quality*	*Minor allele frequency*	*Minor allele effect (% of sd)*^c^	P*-value*	*Location (gene)*	*Best candidate (distance)*	*Comments*
DHEAS	chr7.hg19:g.99075038 T>C	rs148982377T>C	0.927	0.038	−53.1	1.82 × 10^−14^	*ZNF789* (intron)	*CYP3A7* (228 kb)	Published signal for DHEAS.^d^ Same signal as rs34670419 (progesterone)^e^
FAI	chr16.hg19:g.52947630 A>C	rs117145500A>C	0.959	0.063	−35.9	1.50 × 10^−8^	intergenic	*LOC643714* (307 kb)	New signal
FSH	chr11.hg19:g.30226356 T>C	rs11031005T>C	0.978	0.129	−23.2	1.74 × 10^−8^	intergenic	*FSHB* (26 kb)	New signal. Overlaps with LH signal (rs11031002)^e^
LH	chr11.hg19:g.30215261 T>A	rs11031002T>A	0.971	0.121	25.2	3.94 × 10^−9^	intergenic	*FSHB* (37 kb)	New signal. Overlaps with FSH signal (rs11031005)^e^
Oestradiol	chr12.hg19:g.6011490C>A	rs117585797C>A	0.572	0.013	87.1	1.63 × 10^−8^	*ANO2* (intron)	*ANO2* (intronic)	New signal
Progesterone	chr7.hg19:g.99130834G>T	rs34670419G>T	0.927	0.037	−55.6	6.09 × 10^−14^	*ZKSCAN5* (3' UTR)	*CYP3A4* (224 kb) or *CYP3A7* (172 kb)	Published signal for DHEAS.^d^ Same signal as rs148982377 (DHEAS)^e^
Progesterone	chr11.hg19:g.62915346C>G	rs112295236C>G	0.962	0.062	41.0	7.68 × 10^−12^	intergenic	*SLC22A9* (222 kb)	New signal
SHBG	chr17.hg19:g.7574775C>T	rs1641549C>T	0.895	0.239	−28.0	1.21 × 10^−15^	*TP53* (intron)	*SHBG* (38 kb)	Published signal for testosterone^d^

Abbreviations: Chr, chromosome; DHEAS, dehydroepiandrosterone sulphate; FAI, free androgen index; FSH, follicle-stimulating hormone; LH, luteinizing hormone; sd, standard deviation; SHBG, sex hormone binding globulin; UTR, untranslated region.

a Results are for square root of the DHEAS residuals and FAI residuals; inverse rank normal transformed FSH residuals and LH residuals; log_10_ of the oestradiol residuals and progesterone residuals; and ln of the SHBG residuals.

b Details of the reference sequence on which the variant descriptions are based is given in the Supplementary information.

c Minor allele effect sizes are quoted as a per-allele change expressed as a percentage of a standard deviation in the covariate-adjusted transformed residuals.

d Further details regarding the published genetic variants associated with reproductive hormones are given in Supplementary Table 2.

e Effect sizes and *P*-values for each signal in each hormone phenotype are given in Supplementary Table 3.
