# Supplementary material for: Genome-wide association study with 1000 genomes imputation identifies signals for nine sex hormone-related phenotypes
Source: Eur J Hum Genet. 2015 May 27;24(2):284–90. doi: 10.1038/ejhg.2015.102 (PMC4564946; doi:10.1038/ejhg.2015.102)
Supplement: Supplementary Information [file ejhg2015102x1.doc]

**Supplementary Methods.**

*Details of the ElectroChemiLuminescent immunoassays*

**Roche Diagnostics Elecsys 170**

Test Principle : Sandwich Electrochemiluminescence Immunoassay (ECLIA) using Streptavidin-coated Microparticles and with Ruthenium complex

**SHBG**

Measuring range 0.35 to 200 nmol/L

Limit of detection 0.35 nmol/L

Inter assay Precision

1.1% at a level of 15 nmol/L

1.3% at a level of 46 nmol/L

1.7% at a level of 219 nmol/L

Intra assay Precision

1.8% at a level of 14 nmol/L

2.1% at a level of 42 nmol/L

4.0% at a level of 189 nmol/L

**Oestradiol**

Measuring range 18 to 15780 pmol/L

Limit of detection 18 pmol/L

Interassay Precision

1.2% at a level of 130 nmol/L

1.7% at a level of 467 nmol/L

2.0% at a level of 4681 nmol/L

Intra assay Precision

4.7% at a level of 120 nmol/L

2.5% at a level of 472 nmol/L

2.2% at a level of 4693 nmol/L

**Prolactin**

Measuring range 1 to 10000 mU/L

Limit of detection 1 mU/L

Interassay Precision

0.8% at a level of 182 mU/L

1.7% at a level of 598 mU/L

1.1% at a level of 2314 mU/L

Intra assay Precision

1.8% at a level of 288 mU/L

1.4% at a level of 871 mU/L

1.6% at a level of 4477 mU/L

**FSH**

Measuring range 0.1to 200 U/L

Limit of detection 0.1 U/L

Interassay Precision

2.6% at a level of 6.0 U/L

2.8% at a level of 54 U/L

2.5% at a level of 178 U/L

Intra assay Precision

3.6% at a level of 5.3 U/L

3.7% at a level of 46 U/L

4.5% at a level of 229 U/L

**LH**

Measuring range 0.1 to 200 U/L

Limit of detection 0.1 U/L

Interassay Precision

1.2% at a level of 6.2 U/L

0.7% at a level of 92 U/L

0.9% at a level of 164 U/L

Intra assay Precision

2.0% at a level of 5.8 U/L

1.6% at a level of 89 U/L

2.2% at a level of 159 U/L

**DHEA-S**

Measuring range 0.003 to 27 umol/L

Limit of detection 0.003 umol/L

Interassay Precision

3.2% at a level of 2.6 umol/L

2.6% at a level of 10.9 umol/L

2.3% at a level of 21.3 umol/L

Intra assay Precision

2.5% at a level of 2.5 umol/L

2.7% at a level of 10.7 umol/L

2.4% at a level of 20.4 umol/L

**Progesterone**

Measuring range 0.1 to 191 nmol/L

Limit of detection 0.1 nmol/L

Interassay Precision

2.9% at a level of 2.3 nmol/L

1.4% at a level of 9.6 nmol/L

0.9% at a level of 103 nmol/L

Intra assay Precision

4.8% at a level of 2.5 nmol/L

2.8% at a level of 10.0 nmol/L

2.0% at a level of 112 nmol/L

**Testosterone**

Measuring range 0.09 to 52 nmol/L

Limit of detection 0.09 nmol/L

Interassay Precision

14.8% at a level of 0.32 nmol/L

4.1% at a level of 2.42 nmol/L

2.8% at a level of 7.4 nmol/L

2.1% at a level of 45.8 nmol/L

Intra assay Precision

18.1% at a level of 0.32 nmol/L

4.4% at a level of 2.42 nmol/L

3.2% at a level of 7.4 nmol/L

2.5% at a level of 45.8 nmol/L

Supplementary Information on Variants

| **SNP id** | **Hormone** | **Chr-position** | **HGVS name** |
| --- | --- | --- | --- |
| rs148982377T>C | DHEAS | chr7.hg19:g.99075038T>C | NT_007933.15:g. 99075038T>C |
| rs34670419G>T | Progesterone | chr7.hg19:g.99130834G>T | NT_007933.15:g.99130834G>T |
| rs11031002T>A | LH | chr11.hg19:g.30215261T>A | NT_009237.18:g.30215261T>A |
| rs11031005T>C | FSH | chr11.hg19:g.30226356T>C | NT_009237.18:g.30226356T>C |
| rs112295236C>G | Progesterone | chr11.hg19:g.62915346C>G | NT_167190.1:g.62915346C>G |
| rs117585797C>A | Oestradiol | chr12.hg19:g.6011490C>A | NT_009759.16:g.6011490C>A |
| rs117145500A>C | FAI | chr16.hg19:g.52947630A>C | NT_010498.15:g.52947630A>C |
| rs1641549C>T | SHBG | chr17.hg19:g.7574775C>T | NT_010718.16:g.7574775C>T |

**Supplementary Figures and Tables**

*Supplemental Figure 1. Results of hormone GWAS: LocusZoom plots for significant signals (not all genes shown).(Linkage disequilibrium is based on 1000 Genomes Nov 2010 EUR; chr-pos is GRCh37/hg19.)*

*(a) DHEAS – rs148982377, chr7:99,075,038*

*(b) Oestradiol – rs117585797, chr12:6,011,490*

*(c) FAI – rs117145500, chr16:52,947,630*

*(d) FSH – rs11031005, chr11:30,226,356*

*(e) LH – rs11031002, chr11:30,215,261*

*(f) Progesterone, chromosome 7 – rs34670419, chr7:99,130,834*

*(g) Progesterone, chromosome 11 – rs112295236, chr11:62,915,346*

*(h) SHBG – rs1641549, chr17:7,574,775*

*Supplemental Figure 2. LocusZoom plots for the Twins UK FSH and LH GWAS results showing linkage disequilibrium with the known* FSHB *promoter polymorphism (-211 G→T) rs10835638 (chr11.hg19:g.* *30252352 G>T) (shown in purple). (Linkage disequilibrium is based on 1000 Genomes Nov 2010 EUR.)*

*(a) Twins UK FSH GWAS*

*(b) Twins UK LH GWAS*

*Supplementary Table 1. Descriptive statistics for cohort.*

|  |  | % (N=2,913) |  |  |
| --- | --- | --- | --- | --- |
| Sex | Male | 10.17 |  |  |
|  | Female | 89.83 |  |  |
| Menstrual phase | Follicular | 13.86 |  |  |
|  | Luteal | 9.1 |  |  |
|  | Ovulatory | 2.83 |  |  |
|  | Perimenopausal | 11.14 |  |  |
|  | Postmenopausal | 52.9 |  |  |
|  | N/a (male) | 10.17 |  |  |
|  |  |  |  |  |
|  |  | Mean  (standard deviation) | Range | Median  (Lower quartile, upper quartile) |
| Age (years) |  | 53.8 (12.5) | (16,82) | 55 (46,62) |
| Height (m) |  | 1.63 (0.07) | (1.41,2.05) | 1.63 (1.58,1.67) |
| Weight (kg) |  | 70.6 (14) | (37.9,146.5) | 68 (60.5,78) |
| BMI (kg/m^2^) |  | 26.6 (5) | (15.8,53.8) | 25.6 (23.2,29.1) |

*Supplemental Table 2: Summary of values in the Twins UK hormone analyses for published genetic variants associated with reproductive hormones (autosomal variants only) ^2,4,7-10^. Green highlighting indicates effect in same direction for Twins UK study and published, orange highlighting indicates effect in opposite direction.*

*a) DHEAS, FAI, FSH, LH, oestradiol.*

| **Published study** | | | | | **DHEAS** | | **FAI** | | **FSH** | | **LH** | | **Oestradiol** | |
| --- | --- | --- | --- | --- | --- | --- | --- | --- | --- | --- | --- | --- | --- | --- |
| **Chr-position (GRCh37/hg19)** | **Study** | **SNP id** | **Near gene** | **Dir. effect** | **Beta** | **P-value** | **Beta** | **P-value** | **Beta** | **P-value** | **Beta** | **P-value** | **Beta** | **P-value** |
| 10-94485211 | Zhai (DHEAS) | rs2497306 | *HHEX* | + | 3.37E-02 | 1.55E-02 | 3.47E-02 | 1.28E-01 | 2.59E-02 | 3.55E-01 | 1.57E-02 | 5.23E-01 | -7.75E-03 | 6.99E-01 |
| 10-96751270 | Zhai (DHEAS) | rs2185570 | *CYP2C9* | + | 2.63E-02 | 1.95E-01 | 2.08E-02 | 5.35E-01 | -2.91E-02 | 4.76E-01 | -5.81E-02 | 1.04E-01 | 6.01E-03 | 8.37E-01 |
| 15-40360741 | Zhai (DHEAS) | rs7181230 | *BMF* | - | -4.46E-02 | 1.86E-03 | 1.26E-03 | 9.57E-01 | 5.94E-03 | 8.39E-01 | -3.75E-02 | 1.44E-01 | -2.71E-02 | 1.95E-01 |
| 19-48401893 | Zhai (DHEAS) | rs2637125 | *SULT2A1* | + | 8.89E-02 | 1.91E-06 | 8.59E-03 | 7.80E-01 | -2.21E-02 | 5.63E-01 | -6.45E-02 | 5.48E-02 | -6.99E-02 | 1.02E-02 |
| 2-111949327 | Zhai (DHEAS) | rs6738028 | *BCL2L11* | - | -4.94E-02 | 8.10E-04 | -2.05E-02 | 4.00E-01 | 2.68E-02 | 3.69E-01 | 3.24E-02 | 2.15E-01 | -2.13E-02 | 3.16E-01 |
| 7-98957880 | Zhai (DHEAS) | rs740160 | *ARPC1A* | - | 1.45E-02 | 6.59E-01 | 3.37E-02 | 5.40E-01 | -1.82E-02 | 7.88E-01 | 4.20E-02 | 4.75E-01 | 1.06E-01 | 2.84E-02 |
| 7-99118801 | Zhai (DHEAS) | rs11761528 | *ZKSCAN5* | + | **1.42E-01** | **1.79E-09** | 7.82E-02 | 4.78E-02 | -2.05E-02 | 6.70E-01 | 1.07E-02 | 7.98E-01 | 8.35E-02 | 1.52E-02 |
| 7-99489571 | Zhai (DHEAS) | rs17277546 | *TRIM4; CYP3A43* | + | 5.90E-02 | 2.86E-03 | 1.01E-03 | 9.76E-01 | 1.17E-02 | 7.75E-01 | 3.59E-02 | 3.14E-01 | -6.71E-02 | 2.20E-02 |

| **Published study** | | | | | **DHEAS** | | **FAI** | | **FSH** | | **LH** | | **Oestradiol** | |
| --- | --- | --- | --- | --- | --- | --- | --- | --- | --- | --- | --- | --- | --- | --- |
| **Chr-position (GRCh37/hg19)** | **Study** | **SNP id** | **Near gene** | **Dir. effect** | **Beta** | **P-value** | **Beta** | **P-value** | **Beta** | **P-value** | **Beta** | **P-value** | **Beta** | **P-value** |
| 15-51524292 | Chen(FSH) | rs2414095 | *CYP19A1* | + | 4.19E-03 | 7.76E-01 | 1.94E-02 | 4.18E-01 | -3.67E-03 | 9.01E-01 | -1.63E-02 | 5.30E-01 | -7.52E-02 | 3.96E-04 |
| 15-51524292 | Chen (Oestradiol) | rs2414095 | *CYP19A1* | - | 4.19E-03 | 7.76E-01 | 1.94E-02 | 4.18E-01 | -3.67E-03 | 9.01E-01 | -1.63E-02 | 5.30E-01 | -7.52E-02 | 3.96E-04 |
| 15-51617708 | Chen (Oestradiol) | rs2445762 | *CYP19A1* | + | 4.97E-02 | 9.62E-04 | 9.21E-02 | 2.11E-04 | -3.46E-02 | 2.56E-01 | -3.57E-02 | 1.82E-01 | 3.71E-02 | 8.87E-02 |
| 17-7487108 | Chen (SHBG) | rs2075230 | *SHBG* | + | 3.58E-02 | 7.16E-02 | -4.01E-02 | 2.25E-01 | -1.37E-02 | 7.36E-01 | 2.64E-02 | 4.59E-01 | 5.03E-02 | 8.32E-02 |
| 10-65138910 | Coviello (SHBG) | rs7910927 | *JMJD1C* | - | -1.95E-02 | 1.64E-01 | 5.09E-02 | 2.62E-02 | -3.29E-02 | 2.43E-01 | -1.56E-02 | 5.29E-01 | 1.98E-02 | 3.26E-01 |
| 1-107546375 | Coviello (SHBG) | rs17496332 | *PRMT6* | - | -7.18E-03 | 6.24E-01 | 5.48E-05 | 9.98E-01 | 1.26E-02 | 6.72E-01 | -1.78E-02 | 4.95E-01 | -1.30E-02 | 5.40E-01 |
| 12-21331549 | Coviello (SHBG) | rs4149056 | *SLCO1B1* | + | -5.87E-02 | 2.20E-03 | -3.19E-02 | 3.09E-01 | 8.22E-02 | 3.49E-02 | 2.33E-02 | 4.95E-01 | -1.98E-02 | 4.76E-01 |
| 15-96708291 | Coviello (SHBG) | rs8023580 | *NR2F2* | - | -2.11E-02 | 1.71E-01 | 9.66E-03 | 7.04E-01 | -4.17E-02 | 1.90E-01 | -3.43E-02 | 2.18E-01 | -4.57E-02 | 4.35E-02 |
| 17-47445751 | Coviello (SHBG) | rs2411984 | *ZNF652* | - | 4.28E-03 | 7.70E-01 | 5.47E-02 | 2.32E-02 | 2.21E-02 | 4.57E-01 | 3.12E-02 | 2.33E-01 | 5.72E-03 | 7.87E-01 |
| 17-7521915 | Coviello (SHBG) | rs12150660 | *SHBG* | - | 2.60E-03 | 8.69E-01 | 7.82E-02 | 2.55E-03 | 2.05E-02 | 5.21E-01 | 7.75E-03 | 7.82E-01 | -3.66E-02 | 1.06E-01 |

| **Published study** | | | | | **DHEAS** | | **FAI** | | **FSH** | | **LH** | | **Oestradiol** | |
| --- | --- | --- | --- | --- | --- | --- | --- | --- | --- | --- | --- | --- | --- | --- |
| **Chr-position (GRCh37/hg19)** | **Study** | **SNP id** | **Near gene** | **Dir. effect** | **Beta** | **P-value** | **Beta** | **P-value** | **Beta** | **P-value** | **Beta** | **P-value** | **Beta** | **P-value** |
| 2-27742603 | Coviello (SHBG) | rs780093 | *GCKR* | - | 5.40E-03 | 7.05E-01 | -8.72E-03 | 7.09E-01 | 2.08E-02 | 4.68E-01 | 2.14E-02 | 3.95E-01 | -3.08E-02 | 1.32E-01 |
| 2-48646399 | Coviello (SHBG) | rs10454142 | *LHCGR* | + | -2.45E-03 | 8.76E-01 | 1.95E-02 | 4.46E-01 | 6.34E-02 | 4.48E-02 | 6.16E-02 | 2.63E-02 | 1.69E-02 | 4.53E-01 |
| 4-69591782 | Coviello (SHBG) | rs293428 | *UGT2B15* | - | 9.03E-03 | 5.53E-01 | 3.46E-02 | 1.68E-01 | 3.49E-02 | 2.65E-01 | 3.65E-02 | 1.84E-01 | -2.26E-02 | 3.11E-01 |
| 7-97993362 | Coviello (SHBG) | rs3779195 | *BAIAP2L1* | + | -2.49E-02 | 1.73E-01 | -8.96E-02 | 2.82E-03 | -1.30E-02 | 7.27E-01 | -1.42E-02 | 6.64E-01 | 2.37E-02 | 3.72E-01 |
| 8-81461974 | Coviello (SHBG) | rs440837 | *ZBTB10* | - | -1.28E-02 | 4.32E-01 | 8.57E-03 | 7.49E-01 | 5.26E-02 | 1.10E-01 | 3.97E-02 | 1.69E-01 | -6.48E-02 | 5.84E-03 |
| 17-7537792 | Prescott (SHBG) | rs727428 | *SHBG* | - | -4.69E-03 | 7.46E-01 | 6.43E-02 | 6.84E-03 | -1.92E-02 | 5.13E-01 | 6.41E-03 | 8.04E-01 | 5.46E-04 | 9.79E-01 |
| 17-7487108 | Chen (Testosterone) | rs2075230 | *SHBG* | + | 3.58E-02 | 7.16E-02 | -4.01E-02 | 2.25E-01 | -1.37E-02 | 7.36E-01 | 2.64E-02 | 4.59E-01 | 5.03E-02 | 8.32E-02 |
| 10-65337153 | Jin (Testosterone) | rs10822184 | *JMJD1C* | - | -2.17E-02 | 1.25E-01 | 5.16E-02 | 2.57E-02 | -3.04E-02 | 2.87E-01 | -2.19E-02 | 3.82E-01 | 7.17E-03 | 7.25E-01 |
| 17-7537792 | Jin (Testosterone) | rs727428 | *SHBG* | - | -4.69E-03 | 7.46E-01 | 6.43E-02 | 6.84E-03 | -1.92E-02 | 5.13E-01 | 6.41E-03 | 8.04E-01 | 5.46E-04 | 9.79E-01 |
| 17-7521915 | Ohlsson (Testosterone) | rs12150660 | *SHBG* | - | 2.60E-03 | 8.69E-01 | 7.82E-02 | 2.55E-03 | 2.05E-02 | 5.21E-01 | 7.75E-03 | 7.82E-01 | -3.66E-02 | 1.06E-01 |
| 17-7534678 | Ohlsson (Testosterone) | rs6258 | *SHBG* | + | -2.39E-02 | 7.48E-01 | -4.01E-01 | 1.34E-03 | -4.92E-02 | 7.50E-01 | -1.21E-02 | 9.29E-01 | -1.16E-01 | 2.90E-01 |

*b) Progesterone, prolactin, SHBG, testosterone.*

| **Published study** | | | | | **Progesterone** | | **Prolactin** | | **SHBG** | | **Testosterone** | |
| --- | --- | --- | --- | --- | --- | --- | --- | --- | --- | --- | --- | --- |
| **Chr-position (GRCh37/hg19)** | **Study** | **SNP id** | **Near gene** | **Dir. effect** | **Beta** | **P-value** | **Beta** | **P-value** | **Beta** | **P-value** | **Beta** | **P-value** |
| 10-94485211 | Zhai (DHEAS) | rs2497306 | *HHEX* | + | 5.09E-02 | 4.94E-03 | -2.60E-03 | 8.22E-01 | -1.28E-02 | 3.35E-01 | 2.37E-02 | 1.47E-01 |
| 10-96751270 | Zhai (DHEAS) | rs2185570 | *CYP2C9* | + | 1.45E-03 | 9.56E-01 | -2.68E-02 | 1.12E-01 | -1.27E-02 | 5.11E-01 | -8.98E-04 | 9.70E-01 |
| 15-40360741 | Zhai (DHEAS) | rs7181230 | *BMF* | - | 7.74E-04 | 9.67E-01 | 1.36E-02 | 2.59E-01 | -2.91E-02 | 3.47E-02 | -1.81E-02 | 2.85E-01 |
| 19-48401893 | Zhai (DHEAS) | rs2637125 | *SULT2A1* | + | 1.34E-02 | 5.87E-01 | -6.52E-03 | 6.80E-01 | 3.10E-03 | 8.63E-01 | 1.44E-02 | 5.16E-01 |
| 2-111949327 | Zhai (DHEAS) | rs6738028 | *BCL2L11* | - | -5.84E-02 | 2.47E-03 | -4.09E-03 | 7.41E-01 | 7.29E-05 | 9.96E-01 | -1.73E-02 | 3.22E-01 |
| 7-98957880 | Zhai (DHEAS) | rs740160 | *ARPC1A* | - | 1.18E-02 | 7.89E-01 | -1.52E-02 | 5.84E-01 | -3.57E-02 | 2.59E-01 | 4.32E-03 | 9.13E-01 |
| 7-99118801 | Zhai (DHEAS) | rs11761528 | *ZKSCAN5* | + | 1.75E-01 | **3.34E-08** | -1.02E-02 | 6.05E-01 | 1.59E-02 | 4.80E-01 | 1.05E-01 | 2.18E-04 |
| 7-99489571 | Zhai (DHEAS) | rs17277546 | *TRIM4;CYP3A43* | + | 6.32E-02 | 1.72E-02 | 6.39E-03 | 7.03E-01 | 3.09E-02 | 1.08E-01 | 2.90E-02 | 2.32E-01 |
| 15-51524292 | Chen(FSH) | rs2414095 | *CYP19A1* | + | -1.02E-02 | 5.92E-01 | -2.06E-02 | 9.44E-02 | -1.49E-02 | 2.89E-01 | -7.60E-03 | 6.59E-01 |
| 15-51524292 | Chen (Oestradiol) | rs2414095 | *CYP19A1* | - | -1.02E-02 | 5.92E-01 | -2.06E-02 | 9.44E-02 | -1.49E-02 | 2.89E-01 | -7.60E-03 | 6.59E-01 |

| **Published study** | | | | | **Progesterone** | | **Prolactin** | | **SHBG** | | **Testosterone** | |
| --- | --- | --- | --- | --- | --- | --- | --- | --- | --- | --- | --- | --- |
| **Chr-position (GRCh37/hg19)** | **Study** | **SNP id** | **Near gene** | **Dir. effect** | **Beta** | **P-value** | **Beta** | **P-value** | **Beta** | **P-value** | **Beta** | **P-value** |
| 15-51617708 | Chen (Oestradiol) | rs2445762 | *CYP19A1* | + | 4.53E-02 | 2.14E-02 | 1.06E-02 | 4.00E-01 | -1.67E-02 | 2.47E-01 | 7.16E-02 | 6.15E-05 |
| 17-7487108 | Chen (SHBG) | rs2075230 | *SHBG* | + | 3.25E-03 | 9.01E-01 | 7.16E-03 | 6.70E-01 | 7.47E-02 | 9.48E-05 | 5.68E-02 | 1.67E-02 |
| 10-65138910 | Coviello (SHBG) | rs7910927 | *JMJD1C* | - | -1.56E-02 | 3.91E-01 | 1.85E-02 | 1.13E-01 | -4.86E-02 | 2.93E-04 | -1.35E-02 | 4.13E-01 |
| 1-107546375 | Coviello (SHBG) | rs17496332 | *PRMT6* | - | -2.03E-03 | 9.16E-01 | -2.04E-03 | 8.68E-01 | -3.59E-03 | 7.98E-01 | -2.20E-03 | 8.99E-01 |
| 12-21331549 | Coviello (SHBG) | rs4149056 | *SLCO1B1* | + | -3.45E-02 | 1.68E-01 | 2.08E-03 | 8.97E-01 | 9.83E-03 | 5.91E-01 | -2.80E-02 | 2.14E-01 |
| 15-96708291 | Coviello (SHBG) | rs8023580 | *NR2F2* | - | -3.90E-02 | 5.63E-02 | -1.83E-02 | 1.60E-01 | -4.16E-02 | 5.12E-03 | -2.44E-02 | 1.82E-01 |
| 17-47445751 | Coviello (SHBG) | rs2411984 | *ZNF652* | - | -4.06E-03 | 8.33E-01 | -5.62E-03 | 6.48E-01 | -4.70E-02 | 7.97E-04 | -2.22E-03 | 8.98E-01 |
| 17-7521915 | Coviello (SHBG) | rs12150660 | *SHBG* | - | -7.75E-03 | 7.07E-01 | 1.07E-02 | 4.16E-01 | -1.08E-01 | 7.92E-13 | -4.60E-02 | 1.33E-02 |
| 2-27742603 | Coviello (SHBG) | rs780093 | *GCKR* | - | 2.22E-02 | 2.28E-01 | -1.91E-02 | 1.07E-01 | -3.94E-02 | 3.72E-03 | -2.97E-02 | 7.60E-02 |
| 2-48646399 | Coviello (SHBG) | rs10454142 | *LHCGR* | + | -2.47E-02 | 2.28E-01 | 1.37E-02 | 2.95E-01 | -3.69E-02 | 1.38E-02 | -1.70E-02 | 3.56E-01 |
| 4-69591782 | Coviello (SHBG) | rs293428 | *UGT2B15* | - | -1.28E-02 | 5.25E-01 | -1.13E-02 | 3.81E-01 | -1.40E-02 | 3.39E-01 | 1.59E-02 | 3.79E-01 |
| **Published study** | | | | | **Progesterone** | | **Prolactin** | | **SHBG** | | **Testosterone** | |
| **Chr-position (GRCh37/hg19)** | **Study** | **SNP id** | **Near gene** | **Dir. effect** | **Beta** | **P-value** | **Beta** | **P-value** | **Beta** | **P-value** | **Beta** | **P-value** |
| 7-97993362 | Coviello (SHBG) | rs3779195 | *BAIAP2L1* | + | -1.14E-02 | 6.35E-01 | -3.41E-02 | 2.65E-02 | 6.60E-02 | 1.64E-04 | -1.96E-02 | 3.61E-01 |
| 8-81461974 | Coviello (SHBG) | rs440837 | *ZBTB10* | - | 3.14E-03 | 8.82E-01 | -8.55E-04 | 9.50E-01 | -4.88E-02 | 1.70E-03 | -4.08E-02 | 3.34E-02 |
| 17-7537792 | Prescott (SHBG) | rs727428 | *SHBG* | - | -7.50E-03 | 6.93E-01 | 1.27E-02 | 2.95E-01 | -8.67E-02 | 4.76E-10 | -2.99E-02 | 8.11E-02 |
| 17-7487108 | Chen (Testosterone) | rs2075230 | *SHBG* | + | 3.25E-03 | 9.01E-01 | 7.16E-03 | 6.70E-01 | 7.47E-02 | 9.48E-05 | 5.68E-02 | 1.67E-02 |
| 10-65337153 | Jin (Testosterone) | rs10822184 | *JMJD1C* | - | -1.90E-02 | 3.01E-01 | 1.15E-02 | 3.29E-01 | -4.58E-02 | 7.37E-04 | -6.76E-03 | 6.84E-01 |
| 17-7537792 | Jin (Testosterone) | rs727428 | *SHBG* | - | -7.50E-03 | 6.93E-01 | 1.27E-02 | 2.95E-01 | -8.67E-02 | **4.76E-10** | -2.99E-02 | 8.11E-02 |
| 17-7521915 | Ohlsson (Testosterone) | rs12150660 | *SHBG* | - | -7.75E-03 | 7.07E-01 | 1.07E-02 | 4.16E-01 | -1.08E-01 | **7.92E-13** | -4.60E-02 | 1.33E-02 |
| 17-7534678 | Ohlsson (Testosterone) | rs6258 | *SHBG* | + | -3.16E-02 | 7.51E-01 | -4.38E-02 | 4.92E-01 | 4.83E-01 | **1.45E-11** | 5.13E-02 | 5.72E-01 |

Note: There was evidence of consistency of effects for DHEAS (7/8 same direction, *p*=0.04), SHBG (12/13 same direction, *p*=0.002) and testosterone (5/5 same direction, *p*=0.03). No evidence of consistency for oestradiol (2/2 same direction, *p*=0.25) and FSH (0/1 same
direction, *p*=0.5).

*Supplemental Table 3. Effect sizes and p-values for the significant signals in the other hormones in the Twins UK hormone GWAS.*

|  | |  | **Significant signals identified in Twins UK GWAS – hormone and chr-position** | | | | | | | |
| --- | --- | --- | --- | --- | --- | --- | --- | --- | --- | --- |
| **Values of the significant signal in the other Twins UK hormone GWAS results** | | | **DHEAS**  chr7.hg19: g.99075038 T>C,  rs148982377 | **FAI**  chr16.hg19: g.52947630 A>C,  rs117145500 | **FSH**  chr11.hg19: g.30226356 T>C,  rs11031005 | **LH**  chr11.hg19: g.30215261 T>A,  rs11031002 | **Oestradiol**  chr12.hg19: g.6011490 C>A,  rs11758579 | **Progesterone**  chr7.hg19: g.99130834 G>T,  rs34670419 | **Progesterone**  chr11.hg19: g.62915346 C>G,  rs112295236 | **SHBG**  chr17.hg19: g.7574775 C>T,  rs1641549 |
| **DHEAS** | Effect | | -0.255 | **-0.103** | 0.027 | 0.023 | 0.127 | **-0.259** | 0.054 | -0.025 |
|  | P-value | | 1.82E-14 | **4.29E-04** | 1.76E-01 | 2.76E-01 | 9.02E-02 | **1.97E-14** | 5.66E-02 | 1.30E-01 |
| **FAI** | Effect | | -0.093 | -0.276 | 0.02 | 0.021 | 0.11 | -0.101 | 0.082 | 0.064 |
|  | P-value | | 9.79E-02 | 1.50E-08 | 5.48E-01 | 5.51E-01 | 3.73E-01 | 7.61E-02 | 8.20E-02 | 1.89E-02 |
| **FSH** | Effect | | 0.018 | 0.071 | -0.232 | **-0.226** | -0.017 | 0.038 | 0.053 | 0.052 |
|  | P-value | | 7.97E-01 | 2.36E-01 | 1.74E-08 | **1.24E-07** | 9.14E-01 | 5.83E-01 | 3.60E-01 | 1.22E-01 |
| **LH** | Effect | | -0.019 | 0.11 | **0.203** | 0.221 | 0.103 | -0.015 | -0.069 | 0.026 |
|  | P-value | | 7.56E-01 | 3.49E-02 | **1.84E-08** | 3.94E-09 | 4.52E-01 | 8.11E-01 | 1.75E-01 | 3.71E-01 |
| **Oestradiol** | Effect | | -0.071 | -0.023 | -0.048 | -0.045 | 0.624 | -0.084 | -0.02 | -0.065 |
|  | P-value | | 1.47E-01 | 5.81E-01 | 9.75E-02 | 1.40E-01 | 1.63E-08 | 8.99E-02 | 6.21E-01 | 6.63E-03 |
| **Prolactin** | Effect | | 0.005 | -0.034 | -0.004 | 0.001 | 0.066 | 0.006 | 0.028 | 0.02 |
|  | P-value | | 8.49E-01 | 1.62E-01 | 8.06E-01 | 9.66E-01 | 3.03E-01 | 8.30E-01 | 2.42E-01 | 1.62E-01 |
| **Progesterone** | Effect | | **-0.331** | -0.093 | 0.012 | 0.008 | 0.129 | -0.346 | 0.255 | -0.033 |
|  | P-value | | **2.99E-13** | 1.67E-02 | 6.51E-01 | 7.71E-01 | 1.98E-01 | 6.09E-14 | 7.68E-12 | 1.27E-01 |
| **SHBG** | Effect | | -0.085 | 0.121 | -0.018 | -0.017 | 0.066 | -0.079 | -0.021 | -0.127 |
|  | P-value | | 7.85E-03 | **1.51E-05** | 3.55E-01 | 4.04E-01 | 3.63E-01 | 1.52E-02 | 4.51E-01 | **1.21E-15** |
| **Testosterone** | Effect | | **-0.203** | -0.087 | -0.002 | -0.004 | 0.117 | **-0.213** | 0.039 | **-0.071** |
|  | P-value | | **5.39E-07** | 1.39E-02 | 9.22E-01 | 8.78E-01 | 1.85E-01 | **2.33E-07** | 2.44E-01 | **2.73E-04** |

Notes:

Values in bold are significant at *p*<5.00×10^-3^ (*p*<0.05 adjusted for approximately 10 tests per significant signal).

Effect sizes shown are for the minor allele.

*Supplemental Table 4. Effect sizes and p-values in the progesterone GWAS for variants known to be associated with DHEAS from the meta-analysis of Zhai et al^4^. The effects from the Twins UK GWAS are for the effect alleles stated in Zhai et al^4^*.

| **DHEAS variants identified by Zhai et al**^4^ | | | | | | **Values in Twins UK progesterone GWAS analysis** | | |
| --- | --- | --- | --- | --- | --- | --- | --- | --- |
| **Chr.-position (GRCh37/ hg19)** | **SNP** | **Gene** | **Effect allele** | **Effect** | **P-value** | **Effect** | **Standard error** | **P-value** |
| 7-99118801 | rs11761528 | *ZKSCAN5* | T | -0.16 | 3.15×10^-36^ | -0.175 | 0.031 | **3.34×10^-8^** |
| 19-48401893 | rs2637125 | *SULT2A1* | A | -0.09 | 2.61×10^-19^ | -0.013 | 0.025 | 5.87×10^-1^ |
| 15-40360741 | rs7181230 | *BMF* | G | 0.05 | 5.44×10^-11^ | -0.001 | 0.019 | 9.67×10^-1^ |
| 10-94485211 | rs2497306 | *HHEX* | C | -0.04 | 4.64×10^-9^ | -0.051 | 0.018 | **4.94×10^-3^** |
| 10-96751270 | rs2185570 | *CYP2C9* | C | -0.06 | 2.29×10^-8^ | -0.001 | 0.026 | 9.56×10^-1^ |

Chr.=chromosome; DHEAS=dihydroepiandrosterone sulphate; SHBG=sex-hormone binding globulin.

Notes:

The p-value for a binomial sign test of consistency of direction was *p*=0.19.

Values in bold are significant at *p*<0.01 (*p*<0.05 adjusted for five tests).

*Supplemental Table 5. Effect sizes and p-values for the significant progesterone variants identified by the Twins UK GWAS in the data from the DHEAS meta-analysis of Zhai et al^4^ .*

| **Progesterone signal in Twins UK GWAS** | **SNP id** | **Minor allele effect  (% of s.d.)** | **Proxy in Zhai et al^4^** | **r^2^ (SNP and proxy)** | **Gene location** | **P-value in Zhai et al^4^** | **z-score meta-analysis Zhai et al ^4^** |
| --- | --- | --- | --- | --- | --- | --- | --- |
| chr7.hg19: g.99130834G>T | rs34670419 | -55.6 | rs10278040 | 0.58 | Near *CYP3A* genes | **2.34×10^-34^** | -12.2 |
| chr11.hg19:  g.62915346C>G | rs112295236 | 41.0 | rs1939768 | 1 | Near *SLC22A9* | **1.53×10^-4^** | 3.8 |

Notes:

Both progesterone signals (highlighted in bold) are significant at *p*<2.5×10^-2^ (*p*<0.05 adjusted for two tests).

*Supplemental Table 6. Effect sizes and p-values for the significant variants identified by the Twins UK GWAS in the published GWAS of age at menopause^24^.*

| **Significant variant from Twins UK GWAS** | | | | **Value of proxy in age at menopause meta-analysis** | | | | | |
| --- | --- | --- | --- | --- | --- | --- | --- | --- | --- |
| Hormone GWAS | Significant variant | Effect minor allele | P-value | Proxy | Distance of proxy from variant (GRCh37/hg19) | r^2^ (proxy and variant) | Effect minor allele | P-value | Effect in same direction as Twins UK GWAS? |
| DHEAS | rs148982377 | -0.25 | 1.82×10^-14^ | rs10278040 | 66,335 | 0.65 | 0.05 | 4.77×10^-1^ | n |
| FAI | rs117145500 | -0.28 | 1.50×10^-8^ | rs9928588 | 2,464 | 0.54 | 0.11 | 5.05×10^-2^ | n |
| FSH | rs11031005 | -0.23 | 1.74×10^-8^ | rs11031005 | - | 1.00 | 0.23 | **8.30×10^-8^** | n |
| LH | rs11031002 | 0.22 | 3.94×10^-9^ | rs11031002 | - | 1.00 | 0.25 | **3.52×10^-8^** | y |
| Oestradiol | rs117585797 | 0.62 | 1.63×10^-8^ | rs4764574 | 11,786 | 0.23 | 0.06 | 3.79×10^-1^ | y |
| Progesterone  (chr 7) | rs34670419 | -0.35 | 6.09×10^-14^ | rs10278040 | 10,539 | 0.56 | 0.05 | 4.77×10^-1^ | n |
| Progesterone  (chr 11) | rs112295236 | 0.26 | 7.68×10^-12^ | rs1939768 | 637 | 1.00 | 0.03 | 6.88×10^-1^ | y |
| SHBG | rs1641549 | -0.13 | 1.21×10^-15^ | rs1042522 | 4,697 | 0.88 | 0.06 | 9.17×10^-2^ | n |

Note:

Values in bold are significant at *p*<6.25×10^-3^ (*p*=0.05 adjusted for eight tests).

*Supplemental Table 7. Effect sizes and p-values for the significant variants identified by the Twins UK GWAS in the published GWAS of age at menarche^26^.*

| **Significant variant from Twins UK GWAS** | | | | **Value of proxy in age at menarche meta-analysis** | | | | | |
| --- | --- | --- | --- | --- | --- | --- | --- | --- | --- |
| Hormone GWAS | Significant variant | Effect minor allele | P-value | Proxy | Distance of proxy from variant (GRCh37/hg19) | r^2^ (proxy and variant) | Effect minor allele | P-value | Effect in same direction as Twins UK GWAS? |
| DHEAS | rs148982377 | -0.25 | 1.82×10^-14^ | rs10278040 | 66,335 | 0.65 | 0.01 | 5.02×10^-1^ | n |
| FAI | rs117145500 | -0.28 | 1.50×10^-8^ | rs9928588 | 2,464 | 0.54 | -0.01 | 4.12×10^-1^ | y |
| FSH | rs11031005 | -0.23 | 1.74×10^-8^ | rs11031005 | - | 1.00 | 0.04 | **2.74×10^-5^** | n |
| LH | rs11031002 | 0.22 | 3.94×10^-9^ | rs11031002 | - | 1.00 | 0.04 | **4.56×10^-5^** | y |
| Oestradiol | rs117585797 | 0.62 | 1.63×10^-8^ | rs4764574 | 11,786 | 0.23 | -0.03 | 8.78×10^-2^ | n |
| Progesterone  (chr 7) | rs34670419 | -0.35 | 6.09×10^-14^ | rs10278040 | 10,539 | 0.56 | 0.01 | 5.02×10^-1^ | n |
| Progesterone  (chr 11) | rs112295236 | 0.26 | 7.68×10^-12^ | rs1939768 | 637 | 1.00 | -0.01 | 4.01×10^-1^ | n |
| SHBG | rs1641549 | -0.13 | 1.21×10^-15^ | rs1042522 | 4,697 | 0.88 | -0.01 | 4.40×10^-1^ | y |

Notes:

Values in bold are significant at *p*<6.25×10^-3^ (*p*=0.05 adjusted for eight tests).

*Supplemental Table 8. P-values of published menopause variants^24^ in the Twins UK* GWAS.

| **SNP ID** | **Chr-position (GRCh37/ hg19)** | **DHEAS** | **FAI** | **FSH** | **LH** | **Oestradiol** | **Progesterone** | **Prolactin** | **SHBG** | **Testosterone** |
| --- | --- | --- | --- | --- | --- | --- | --- | --- | --- | --- |
| rs4246511 | 1-39380385 | 8.22E-01 | 6.39E-01 | 1.10E-01 | 4.15E-01 | 4.21E-01 | 5.07E-01 | 7.76E-01 | 2.85E-01 | 6.85E-01 |
| rs2303369 | 2-27715416 | 7.72E-01 | 5.59E-01 | 3.57E-01 | 2.29E-01 | 1.45E-01 | 4.35E-01 | 4.96E-01 | 3.81E-01 | 1.18E-01 |
| rs10183486 | 2-171990971 | 7.91E-01 | 8.85E-01 | 7.47E-01 | 7.77E-01 | 7.32E-01 | 3.18E-01 | 7.63E-01 | 5.85E-01 | 4.17E-02 |
| rs1635501 | 1-242040775 | 7.29E-01 | 3.17E-01 | 4.79E-01 | 5.77E-01 | 8.60E-01 | 1.11E-01 | 5.81E-01 | 8.15E-01 | 5.25E-01 |
| rs4693089 | 4-84373622 | 2.31E-01 | 2.26E-01 | 2.86E-01 | 5.18E-02 | 5.48E-01 | 4.71E-01 | 2.85E-03 | 9.74E-01 | 7.91E-01 |
| rs365132 | 5-176378574 | 3.20E-01 | 4.55E-01 | 9.96E-01 | 9.62E-01 | 9.74E-01 | 6.35E-01 | 9.88E-01 | 7.01E-01 | 5.09E-01 |
| rs2153157 | 6-10897488 | 3.83E-01 | 8.58E-01 | 9.07E-01 | 5.63E-01 | 4.51E-01 | 4.77E-01 | 1.00E+00 | 8.98E-01 | 6.35E-01 |
| rs1046089 | 6-31602967 | 9.27E-01 | 3.62E-01 | 8.86E-01 | 1.68E-01 | 4.75E-01 | 1.99E-01 | 7.79E-02 | 6.88E-01 | 4.01E-01 |
| rs2517388 | 8-37977732 | 4.31E-01 | 1.08E-01 | 6.15E-01 | 4.77E-01 | 2.38E-01 | 8.21E-01 | 3.57E-01 | 3.36E-01 | 9.02E-02 |
| rs12294104 | 11-30382899 | 6.14E-01 | 8.73E-01 | **3.02E-07** | **6.25E-07** | 1.53E-01 | 7.61E-01 | 6.44E-01 | 4.01E-01 | 8.88E-01 |
| rs2277339 | 12-57146069 | 1.32E-01 | 2.39E-02 | 8.97E-01 | 9.59E-01 | 3.96E-01 | 6.15E-02 | 8.11E-01 | 1.60E-02 | 6.79E-01 |
| rs4886238 | 13-61113739 | 2.43E-01 | 7.13E-01 | 3.87E-02 | 2.35E-01 | 3.98E-01 | 8.88E-02 | 5.21E-01 | 7.63E-02 | 6.42E-01 |
| rs2307449 | 15-89863928 | 1.59E-01 | 9.56E-01 | 7.80E-01 | 2.82E-01 | 5.51E-01 | 3.02E-01 | 5.85E-01 | 5.12E-01 | 9.31E-01 |
| rs10852344 | 16-12016919 | 4.53E-01 | 2.98E-01 | 7.91E-02 | 6.62E-02 | 4.14E-01 | 1.99E-01 | 2.54E-01 | 5.19E-01 | 1.22E-01 |
| rs11668344 | 19-55833664 | 6.58E-01 | 5.96E-01 | 2.88E-01 | 6.97E-01 | 6.09E-01 | 2.30E-01 | 8.61E-02 | 9.81E-01 | 1.66E-01 |
| rs12461110 | 19-56320663 | 6.41E-01 | 8.44E-01 | 2.63E-01 | 2.92E-01 | 4.60E-01 | 3.33E-01 | 1.69E-01 | 9.95E-01 | 8.23E-01 |
| rs16991615 | 20-5948227 | 5.02E-01 | 2.82E-01 | 7.31E-01 | 5.26E-01 | 1.05E-01 | 2.55E-01 | 7.16E-01 | 7.28E-01 | 3.07E-02 |

Note:

Values in bold are significant at *p*<3.3×10^-4^, calculated on the basis of nine tests at each of 17 published SNPs.

*Supplemental Table 9. P-values of published menarche variants^26^ in the Twins UK* GWAS.

| **SNP ID** | **Chr-position (GRCh37/hg19)** | **DHEAS** | **FAI** | **FSH** | **LH** | **Oestradiol** | **Progesterone** | **Prolactin** | **SHBG** | **Testosterone** |
| --- | --- | --- | --- | --- | --- | --- | --- | --- | --- | --- |
| rs466639 | 1-165394882 | 7.89E-02 | 1.26E-01 | 1.03E-01 | 2.62E-01 | 7.12E-01 | 6.75E-01 | 9.27E-01 | 1.71E-01 | 6.20E-04 |
| rs633715 | 1-177852580 | 1.05E-01 | 2.89E-01 | 9.31E-01 | 5.02E-01 | 8.33E-01 | 9.75E-02 | 9.85E-01 | 1.92E-01 | 2.55E-02 |
| rs2947411 | 2-614168 | 9.88E-01 | 6.16E-01 | 5.64E-01 | 4.49E-01 | 7.03E-01 | 2.64E-01 | 4.96E-01 | 7.13E-01 | 4.44E-01 |
| rs17268785 | 2-56592083 | 9.62E-01 | 6.05E-01 | 6.92E-01 | 7.77E-01 | 8.17E-01 | 1.98E-01 | 6.29E-01 | 7.17E-01 | 7.59E-01 |
| rs17188434 | 2-157096776 | 9.45E-01 | 6.12E-01 | 2.94E-01 | 6.02E-01 | 4.46E-01 | 3.94E-01 | 6.45E-01 | 8.89E-01 | 4.08E-01 |
| rs12617311 | 2-199632565 | 7.79E-01 | 8.80E-01 | 2.06E-01 | 4.13E-01 | 3.72E-01 | 3.62E-01 | 9.15E-01 | 3.04E-01 | 5.92E-01 |
| rs7617480 | 3-49210732 | 8.63E-01 | 7.90E-01 | 8.54E-01 | 8.20E-01 | 2.40E-01 | 1.03E-01 | 7.20E-01 | 7.31E-01 | 5.30E-01 |
| rs6762477 | 3-50093209 | 9.83E-01 | 7.05E-01 | 1.70E-01 | 1.05E-01 | 3.69E-01 | 9.32E-01 | 8.70E-01 | 1.10E-01 | 1.86E-01 |
| rs7642134 | 3-86916882 | 1.10E-01 | 9.50E-01 | 2.34E-03 | 2.55E-01 | 8.07E-01 | 6.80E-02 | 1.06E-01 | 4.88E-01 | 7.19E-01 |
| rs6438424 | 3-117574822 | 8.96E-01 | 1.30E-01 | 7.05E-02 | 2.07E-01 | 4.38E-01 | 9.49E-01 | 1.28E-01 | 5.17E-01 | 5.65E-01 |
| rs6439371 | 3-132610752 | 1.81E-01 | 9.60E-02 | 3.93E-01 | 7.49E-01 | 7.41E-01 | 1.13E-01 | 6.17E-01 | 5.90E-01 | 8.20E-01 |
| rs2002675 | 3-185629568 | 6.99E-02 | 8.17E-01 | 6.25E-01 | 3.59E-01 | 7.10E-01 | 6.08E-01 | 8.94E-01 | 6.98E-01 | 5.24E-01 |
| rs13187289 | 5-133849177 | 6.49E-01 | 2.52E-01 | 9.41E-01 | 2.92E-01 | 9.92E-01 | 3.53E-01 | 2.16E-02 | 8.97E-01 | 7.02E-02 |
| rs4840086 | 6-100208438 | 1.35E-01 | 3.62E-01 | 2.93E-01 | 2.41E-01 | 4.59E-01 | 7.57E-02 | 6.36E-01 | 7.70E-01 | 1.83E-01 |
| rs7759938 | 6-105378954 | 6.83E-01 | 5.43E-02 | 9.97E-01 | 8.83E-01 | 4.89E-01 | 1.50E-01 | 7.82E-01 | 2.67E-02 | 9.63E-01 |
| rs1361108 | 6-126767600 | 4.97E-01 | 4.44E-01 | **1.50E-04** | 4.80E-02 | 6.83E-01 | 2.86E-01 | 1.13E-02 | 5.24E-01 | 6.21E-01 |
| rs1079866 | 7-41470093 | 3.88E-01 | 1.14E-01 | 4.70E-02 | 7.54E-02 | 6.40E-01 | 7.53E-01 | 1.40E-03 | 6.17E-01 | 5.76E-02 |
| rs7821178 | 8-78093837 | 4.98E-01 | 8.63E-01 | 7.14E-01 | 5.39E-01 | 8.92E-01 | 6.43E-02 | 8.77E-01 | 2.90E-01 | 4.13E-01 |
| rs2090409 | 9-108967088 | 9.40E-01 | 5.81E-01 | 3.13E-01 | 4.62E-01 | 5.32E-01 | 6.52E-01 | 8.12E-01 | 3.39E-01 | 8.03E-01 |
| **SNP ID** | **Chr-position (GRCh37/hg19)** | **DHEAS** | **FAI** | **FSH** | **LH** | **Oestradiol** | **Progesterone** | **Prolactin** | **SHBG** | **Testosterone** |
| rs10980926 | 9-114293634 | 9.30E-01 | 1.09E-01 | 4.47E-01 | 5.03E-02 | 8.17E-01 | 3.83E-01 | 9.77E-02 | 3.87E-01 | 6.87E-01 |
| rs4929923 | 11-8639200 | 8.20E-01 | 4.03E-01 | 1.66E-01 | 3.80E-02 | 6.04E-01 | 2.67E-01 | 2.73E-01 | 7.43E-02 | 7.98E-01 |
| rs900145 | 11-13293905 | 1.96E-01 | 1.46E-01 | 7.90E-01 | 8.02E-01 | 8.03E-01 | 3.74E-01 | 1.12E-01 | 2.30E-01 | 4.71E-02 |
| rs10899489 | 11-78095373 | 1.08E-01 | 8.43E-01 | 7.48E-01 | 7.77E-01 | 4.39E-01 | 5.12E-01 | 9.94E-01 | 6.40E-01 | 6.74E-01 |
| rs6589964 | 11-122870683 | 6.40E-01 | 1.00E-01 | 6.77E-01 | 7.16E-01 | 1.05E-01 | 3.95E-01 | 6.61E-01 | 2.72E-01 | 3.34E-01 |
| rs6575793 | 14-101032217 | 2.64E-01 | 9.76E-01 | 9.35E-01 | 2.08E-01 | 3.53E-01 | 6.00E-01 | 4.96E-01 | 9.15E-01 | 8.45E-01 |
| rs1659127 | 16-14388305 | 7.49E-01 | 7.42E-01 | 5.67E-01 | 9.20E-02 | 7.46E-01 | 3.55E-01 | 2.06E-01 | 6.54E-01 | 9.15E-01 |
| rs9939609 | 16-53820527 | 8.64E-01 | 2.31E-01 | 3.62E-01 | 9.54E-01 | 1.44E-01 | 8.31E-01 | 2.70E-01 | 4.69E-01 | 2.80E-01 |
| rs1364063 | 16-69588572 | 2.90E-01 | 4.99E-01 | 5.11E-01 | 6.59E-01 | 8.67E-01 | 2.55E-01 | 2.08E-01 | 3.66E-01 | 9.25E-01 |
| rs9635759 | 17-49613785 | 9.13E-01 | 8.58E-03 | 8.00E-01 | 2.97E-01 | 3.10E-01 | 5.14E-01 | 4.91E-01 | 2.10E-01 | 1.02E-01 |
| rs1398217 | 18-44752238 | 6.89E-01 | 7.47E-01 | 1.12E-01 | 9.24E-01 | 5.92E-01 | 7.96E-01 | 1.14E-01 | 7.23E-01 | 4.53E-01 |
| rs10423674 | 19-18817903 | 5.09E-01 | 6.90E-01 | 2.68E-01 | 1.52E-01 | 6.30E-01 | 1.97E-01 | 9.76E-01 | 1.27E-01 | 1.80E-01 |
| rs852069 | 20-17122593 | 6.03E-01 | 8.10E-01 | 5.38E-01 | 4.30E-01 | 2.43E-01 | 9.58E-01 | 6.91E-01 | 6.71E-01 | 6.39E-01 |

Note:

Values in bold are significant at *p*<1.74×10^-4^, calculated on the basis of nine tests at 32 published SNPs.

*Supplemental Table 10. Candidate genes and expression qualitative trait loci (eQTL)* associated with the significant signals.

| **Hormone** | **Chr-position** | **SNP id** | **P-value** | **Location of SNP** | **eQTL (Proxy for signal r^2^>0.8 with associations)** | **Other genes within 300kb from start/end of gene** |
| --- | --- | --- | --- | --- | --- | --- |
| DHEAS | chr7.hg19:g.99075038T>C | rs148982377T>C | 1.82×10^-14^ | *ZNF789* | N/a^1^ | *ARPC1A*, *ARPC1B*, *ATP5J2*, *ATP5J2-PTCD1*, *BUD31*, *CPSF4*, *CYP3A4*, *CYP3A5*, *CYP3A7*, *CYP3A7-CYP3AP1*, *FAM200A*, *KPNA7*, *LOC100289187*, *MHY* ^2^, *PDAP1*, *PTCD1*, *ZKSCAN5*, *ZNF394* ^2^, *ZNF655*, *ZNF789*, *ZSCAN25* |
| FAI | chr16.hg19:g.52947630A>C | rs117145500A>C | 1.50×10^-8^ | intergenic | N/a^1^ | *CHD9*, *LOC643714* |
| FSH | chr11.hg19:g.30226356T>C | rs11031005T>C | 1.74×10^-8^ | intergenic | rs11031005 – No eQTL associations | *ARL14EP* ^2^, *FSHB*, *KCNA4*, *MPPED2* |
| LH | chr11.hg19:g.30215261T>A | rs11031002T>A | 3.94×10^-9^ | intergenic | rs11031002 – No eQTL associations | *ARL14EP* ^2^, *FSHB*, *KCNA4*, *MPPED2* |
| Oestradiol | chr12.hg19:g.6011490C>A | rs117585797C>A | 1.63×10^-8^ | *ANO2* | N/a^1^ | *CD9*, *VWF* |
| Progesterone | chr7.hg19:g.99130834G>T | rs34670419G>T | 6.09×10^-14^ | *ZKSCAN5* | N/a^1^ | *ARPC1A*, *ARPC1B*, *ATP5J2*, *ATP5J2-PTCD1*, *BUD31*, *CPSF4*, *CYP3A4*, *CYP3A43*, *CYP3A5*, *CYP3A7*, *CYP3A7-CYP3AP1*, *FAM200A*, *LOC100289187*, *MHY* ^2^, *PDAP1*, *PTCD1*, *ZKSCAN5*, *ZNF394* ^2^, *ZNF655*, *ZNF789*, *ZSCAN25* |
| Progesterone | chr11.hg19:g.62915346C>G | rs112295236C>G | 7.68×10^-12^ | intergenic | rs1939768 – No eQTL associations | *CHRM1*, *HRASLS5*, *MIR3680-1* ^2^, *MIR3680-2* ^2^, *SLC22A10*, *SLC22A24*, *SLC22A25*, *SLC22A6*, *SLC22A8*, *SLC22A9*, *SLC3A2* |
| SHBG | chr17.hg19:g.7574775C>T | rs1641549C>T | 1.21×10^-15^ | *TP53* | rs1042522 – *EFNB3* in adipose (*p*=9.9E-12) and skin (*p*=2.72E-05) | *ATP1B2*, *C17orf61-PLSCR3*, *C17orf74*, *CD68*, *CHD3*, *CHRNB1*, *CYB5D1*, *DNAH2*, *EFNB3*, *EIF4A1*, *FGF11*, *FXR2*, *KDM6B2*, *LSMD12*, *MPDU1*, *NLGN2*, *PLSCR3*, *POLR2A*, *RPL29P22*, *SAT2*, *SENP3*, *SENP3-EIF4A1*, *SHBG*, *SLC35G6*, *SLC35G6*, *SNORA482*, *SNORA672*, *SNORD102*, *SOX15*, *SPEM12*, *TMEM1022*, *TMEM256*, *TMEM88*, *TNFSF12*, *TNFSF12-TNFSF13*, *TNFSF13*, *TNK1*, *TP53*, *WRAP53*, *ZBTB4* |

Notes:

^1^N/a = Best proxy r^2^<0.8;

^2^ Provisional.

*Supplemental Table 11. Values of the known FSHB promoter polymorphism (-211 G→T) rs10835638 (chr11.hg19:g. 30252352 G>T) in the Twins UK FSH and LH GWAS results*.

| **Hormone** | **Imputation quality** | **Effect allele frequency** | **Effect** | **P-value** |
| --- | --- | --- | --- | --- |
| FSH | 0.983 | 0.857 | 0.209 | 2.31×10^-7^ |
| LH | 0.983 | 0.857 | -0.207 | 4.84×10^-9^ |
